# Exome sequencing in 90 children with developmental delay: a single-center experience

**DOI:** 10.3389/fgene.2024.1505254

**Published:** 2024-11-29

**Authors:** Oksana Boyarchuk, Liubov Volianska, Olena Smashna, Halyna Makukh

**Affiliations:** ^1^ Department of Children’s Diseases and Pediatric Surgery, I.Horbachevsky Ternopil National Medical University, Ternopil, Ukraine; ^2^ Department of the Research and Biotechnology, Scientific Medical Genetic Center LeoGENE, Lviv, Ukraine

**Keywords:** developmental delay, whole exome sequencing, WES, autism spectrum disorders, epilepsy

## Abstract

**Introduction:**

Developmental delay (DD) in children is often caused by genetic abnormalities, which are challenging to diagnose due to the vast genetic variability.

**Methods:**

This study presents a detailed analysis of whole-exome sequencing (WES) on 90 children with DD at a single clinical center.

**Results:**

We identified pathogenic or likely pathogenic variants in 27.8% of cases, with 7.8% revealing variants of uncertain significance (VUS). Among the positive findings, 21 (84.0%) corresponded to the main clinical manifestations in patients, and 4 (16.0%) secondary findings provided new insights into the patient’s conditions. Positive and inconclusive cases led to a revision of the diagnosis or management plan in 34.4% of cases. The positive genetic result in children with Developmental delay was higher in the presence of epilepsy or seizures (odds ratio – 5.4444; 95% CI 2.0176 to 14.6918; *p* = 0.0008) and more than 3 dysmorphic features (odds ratio – 7.1739; 95% CI 1.7791 to 28.9282; *p* = 0.0056). Variants compatible with the clinical manifestations were identified in 11.9% of children with autistic spectrum disorders.

**Conclusion:**

Our findings emphasize the utility of WES in clinical diagnostics, offering significant insights into patient management and potentially guiding therapeutic decisions.

## Introduction

Developmental delay (DD) is a significant concern in pediatrics, potentially impacting a child’s future development (Choo et al., 2019). DD is relatively common, with a prevalence ranging from 5% to 15% among children under the age of five (Vitrikas et al., 2017; Oberklaid and Efron, 2005). DD is defined as the failure of a child to achieve developmental milestones expected for their age. These delays can manifest in motor skills, speech and language, social-emotional, and cognitive development (Vitrikas et al., 2017). DD may affect a single developmental area or multiple areas of a child’s functioning (Choo et al., 2019). When a young child experiences substantial delays in two or more of these areas, it is referred to as global DD (Shevell et al., 2003).

The causes of DD are varied and can occur during the prenatal, perinatal, or postnatal periods (Choo et al., 2019). Prenatal causes frequently include cerebral dysgenesis, maternal infections, exposure to drugs and toxins, and genetic or hereditary factors. Perinatal causes may involve prematurity, perinatal asphyxia, and metabolic disorders. Postnatal causes of DD can include trauma, infections, anoxia, and deprivation of food or environmental stimuli (Choo et al., 2019).

The precise genetic causes of DD can be difficult to determine due to their heterogeneous nature. However, recent advancements in genomic technologies, particularly whole-exome sequencing (WES), have transformed the diagnosis of genetic disorders by simultaneously identifying pathogenic variants across thousands of genes (Bowling et al., 2017; Gerik-Celebi et al., 2023; Zhang et al., 2024). Despite these breakthroughs, the clinical application of WES in routine diagnostics, especially in resource-limited settings, remains insufficiently explored. In Ukraine, NGS technologies are not readily available, and usually patients' families cover the cost of genetic research outside the country.

This study aims to assess the diagnostic yield of WES in a cohort of 90 children with developmental delays and to identify the most significant clinical features associated with genetically determined DD.

## Materials and methods

### Study participants

The study included 90 children with DD referred to the regional center for genetic evaluation. The research consisted of two cohorts. The first cohort included 40 children with DD enrolled from 1 October 2020, to 31 September 2021 (1 year), under a grant received from the 3billion (South Korea) for WES. The second cohort consisted of 50 patients enrolled from 7 December 2022, to 7 April 2023 (4 months), as part of a second grant from the 3billion. All children were under the care of a neurologist or psychiatrist due to DD and were referred for genetic testing. The inclusion criteria were as follows: phenotypes and symptoms in children, including intellectual disability or developmental delay, neurodevelopmental delay, neuromuscular disorder or white matter disease, inborn error of metabolism, and age up to 18 years. The study followed the principles of the 1975 Declaration of Helsinki (amended in 2000) and was approved by the Ethics Committee of I. Horbachevsky Ternopil National Medical University. Informed consent was obtained from all participants’ guardians.

### Whole-exome sequencing

High molecular weight genomic DNA was extracted from patient’s whole blood-EDTA, or buccal swab samples using QIAamp DNA Blood Mini Kit (Qiagen) and AccuBuccal DNA Preparation Kit (Accugene), respectively. WES was performed following the CAP/CLIA validated standard operating protocol. Exome capture was performed using xGen Exome Research Panel v2 (Integrated DNA Technologies, Coralville, Iowa, United States). Sequencing was performed using the NovaSeq 6,000 platform (Illumina, San Diego, CA, United States) as 150 bp paired-end reads. Sequencing data were aligned to either GRCh37/hg19 human reference genome using BWA-MEM and processed for variant calling by GATK v4.2.14 (Van der Auwera et al., 2013; Li and Durbin, 2009). Copy number variants (CNVs) were called using 3bCNV, and tool developed by 3billion that uses depth-of-coverage information of each exon. Variants were annotated, filtered, and classified using EVIDENCE v4 which incorporates Ensembl Variant Effect Predictor (VEP) for annotation and the American College of Medical Genetics and Genomics (ACMG) guideline for variant classification (Richards et al., 2015; Seo et al., 2020; McLaren et al., 2016). The splicing prediction was performed using spliceAI (Jaganathan et al., 2019). The filtered and classified variant list was than manually reviewed by medical geneticists and physicians. The most likely variants that can explain the patient’s phenotype were selected for reporting.

3billion has internally validated that variants (SNV/INDEL) with ‘quality score > 250 and allele fraction > 0.3 (heterozygous), > 0.95 (homozygous) and read depth > 10’ does not require additional validation by Sanger sequencing. This validation process is approved by CAP/CLIA. For variants that do not meet the criteria, Sanger sequencing was performed.

3billion uses internally developed software, GEBRA. The variants are associated with genes/diseases that are relevant. The software leverages OMIM, and other publicly available disease databases including publications from PUBMED to provide up-to-date disease information.

The variants are sorted and arranged by: classification by EVIDENCE and the similarity between the given symptoms/phenotypes of the patient and the disease that the variant is associated with. Then, geneticists review, other details. Other additional features (e.g., gene panel) are included in the software. Multiple panels from different sources (e.g., Genomics England PanelApp) are included.

### Statistical analysis

Qualitative variables are presented as absolute frequencies and percentages, while quantitative variables are expressed as mean and standard deviation (SD). Statistical comparisons between cohorts and groups were conducted using chi-squared tests and t-tests, with the significance level set at *p* < 0.05. The odds ratio (OR) and 95% confidence intervals were calculated to assess the influence of significant factors on positive WES results. Only statistically significant features were included in this analysis. Statistical analysis was performed using STATISTICA 10.

## Results

### Patient characteristics

The cohort consisted of 90 children, with a mean age of 6.33 ± 4.69 years, 61 (67.8%) of whom were male (Table 1). There was no difference in age between the patient cohorts, although the second cohort had a higher proportion of boys. All patients had DD of varying degrees and in different areas of development. The most common issue was speech and language delay (83.3%), followed by intellectual disability of varying severity (72.2%). Behavioral problems were observed less frequently (44.4%), including attention deficit hyperactivity disorder (ADHD) in 24.4% of children. Autism spectrum disorders (ASD) were present in 46.6% of patients, and epilepsy or seizures in 27.8%. Facial dysmorphism was observed in 38.9%. Other symptoms were present in 61.1% of the cohort, with the most common being skeletal deformities (in 17 children – 18.9%), hydrocephalus (in 7 children – 7.8%), eczema (in 8 children – 8.9%), hypotonia (in 17 children – 18.9%), and recurrent infections (in 6 children – 6.7%). A positive family history of DD was noted in one-third of the patients.

**TABLE 1:** Baseline and clinical characteristics and WES results of the study population.

Characteristic	Total	First cohort	Second cohort	*p*
n	90	40	50	
Age, m ± SD	6.33 ± 4.69	6.79 ± 5.29	5,96 ± 4.17	>0.05
	n (%)	n (%)	n (%)	
Male	61 (67.8)	23 (57.5)	38 (76.0)	**0.0282**
Female	29 (32.2)	17 (42.5)	12 (24.0)	**0.0282**
WES results				
Positive	25 (27.8)	19 (47.5)	6 (12.0)	**<0.0001**
Inconclusive	7 (7.8)	4 (10.0)	3 (6.0)	0.4814
Negative	58 (64.4)	17 (42.5)	41 (82.0)	**0.0001**
Compatible with clinical manifestations	27 (30.0)	19 (47.5)	8 (16.0)	**0.0012**
Secondary findings	5 (5.6)	4 (10.0)	1 (2.0)	0.0997
Clinical feature				
Failure to thrive	14 (15.6)	10 (25.0)	4 (8.0)	**0.0270**
Speech and language delay	75 (83.3)	27 (67.5)	48 (96.0)	**0.0003**
Intellectual disability	65 (72.2)	24 (60.0)	41 (82.0)	**0.0206**
Behavior problems	40 (44.4)	14 (35.0)	26 (65.0)	0.1068
ADHD	22 (24.4)	5 (12.5)	17 (34.0)	**0.0184**
Autism spectrum disorders (ASD)	42 (46.6)	9 (22.5)	33 (62.1)	**<0.0001**
Epilepsy or seizure	25 (27.8)	15 (37.5)	10 (20.0)	0.0655
Facial dysmorphism	35 (38.9)	20 (50.0)	15 (30.0)	0.0531
Microcephaly	9 (10.0)	3 (7.5)	6 (12.0)	0.4795
Macrocephaly	4 (4.4)	2 (5.0)	2 (4.0)	0.8191
More than 3 dysmorphic features	12 (13.3)	7 (17.5)	5 (10.0)	0.2983
Other symptoms	55 (61.1)	31 (77.5)	24 (48.0)	**0.0043**
Family history	30 (33.3)	14 (35.0)	16 (32.0)	0.7642

SD, standard deviation, VUS–variant of uncertain significance, ADHD, attention deficit hyperactivity disorder; statistically significant values are highlighted in bold.

### WES results

WES identified pathogenic or likely pathogenic variants in 25 of the 90 patients (27.8%). In addition, 7 patients (7.8%) had inconclusive variants, while 58 patients (64.4%) had no pathogenic variants identified. Among the positive findings, 21 (84.0%) corresponded to the main clinical manifestations in patients, and 4 (16.0%) secondary findings provided new insights into the patient’s conditions. All identified variants and their clinical relevance are shown in Supplementary file, Supplementary Table S1. Among the positive results, there were 4 homozygous variants inherited in an autosomal recessive manner, while the rest were heterozygous variants with autosomal dominant inheritance. One child was found to have two variants - *SMC3* and *MT-TL1* in mitochondrial DNA, which are associated with Cornelia de Lange syndrome 3 and MELAS. The child exhibited symptoms of both syndromes. The cohort included two siblings who were found to have a very rare condition, interferon regulatory factor 2 binding protein-like–related disorder (*IRF2BPL*–related disorder) (Shelkowitz et al., 2019), with the boy having severe impairments and the girl having much milder symptoms. In two patients, a pathogenic and a new likely pathogenic variant were identified in the *ZMYND11* gene, which is associated with intellectual developmental disorder.

Among the inconclusive variants, 4 were VUS, 1 was likely pathogenic (*KDM5C* gene, c.1762C > T, *p*. Gln588Ter variant), 1 was pathogenic (*TNFRSF13B*, c.542C > A, *p*. Ala81GLu), and there was a 15q11.2q12q13.1 duplication as the test was not validated to identify copy number variants. All VUS were consistent with the clinical manifestations of the diseases. Patients with Coffin-Siris syndrome were described in a recently published article (Boyarchuk et al., 2024). The likely pathogenic variant in the *KDM5C* gene was consistent with Intellectual disability, X-linked, syndromic, Claes Jensen type, which is an X-linked recessive disorder. A heterozygous female might be affected due to skewed X-chromosome inactivation or X-chromosomal abnormalities. Additional genetic testing was considered to establish a genetic diagnosis. A heterozygous pathogenic variant identified in the *TNFRSF13B* gene is associated with autosomal recessive common variable immunodeficiency (OMIM: 240500). Only one heterozygous variant was identified, and the molecular diagnosis is challenging because most of these diseases are inherited in an autosomal recessive manner. Other genetic testing may identify a second variant, such as a deletion, duplication, or deep intronic variant, that is not detectable by this test. However, there is growing evidence suggesting that the disease can manifest even as a heterozygous (Castigli et al., 2005). Chromosomal abnormalities were identified in 3 children (2 duplications of chromosome 15 and 1 deletion of chromosome 22).

Overall, in 81.3% of positive and inconclusive cases, the identified genetic variants correlated with the primary clinical features, leading to a revision of the diagnosis or management plan in 11 out of 32 cases (34.4%). Additionally, in 15 out of 32 cases (46.9%), the genetic diagnosis partially influenced patient management (enabling treatment adjustments, avoiding unnecessary medications and procedures), although it did not significantly impact the disease outcomes. Secondary findings (12.5%) implicated future health monitoring. The WES results showed a significantly higher percentage of positive findings in the first cohort (47.5% vs 12.0%, *p* < 0.0001). Therefore, we compared the symptoms present in children from the first and second cohorts (Table 1). In the first cohort, failure to thrive (25.0% vs 8.0%, *p* = 0.0270) and other symptoms (77.5% vs 48.0%, *p* = 0.0043) were more common. A trend toward more frequent occurrences of epilepsy or seizures (*p* = 0.0655) and facial dysmorphism (*p* = 0.0531) also was observed. Conversely, in the second cohort, speech and language delay (96.0% vs 67.6%, *p* = 0.0003), intellectual disability (82.0% vs 60.0%, *p* = 0.0206), ASD (62.1% vs 22.5%, *p* < 0.0001), and ADHD (34.0% vs 12.5%, *p* = 0.0184) were significantly more frequent. No differences were observed for other signs.

### Comparison of positive with negative cases

To identify the features that may have most influenced positive outcomes, we compared the observed signs in patients with positive results and inconclusive results (positive cases) correlated with clinical data, as well as in children with negative results (negative cases) (Table 2).

**TABLE 2:** Comparison of baseline characteristics and main symptoms in positive (positive and inconclusive results) and negative cases (negative results).

Characteristic	Positive cases	Negative cases	*p*
n	32	58	
Age, years, m ± SD	7.54 ± 5.38	5,66 ± 4.17	0.0696
	n (%)	n (%)	
Male	17 (53.1)	44 (75.9)	**0.0271**
Female	15 (46.9)	14 (24.1)	**0.0271**
Failure to thrive	7 (21.9)	7 (12.1)	0.2192
Speech and language delay	24 (75.0)	51 (87.9)	0.1151
Intellectual disability	23 (71.9)	42 (72.4)	0.9564
Behavior problems	11 (34.4)	29 (50.0)	0.1533
ADHD	6 (18.8)	16 (27.6)	0.3505
Autism spectrum disorders (ASD)	6 (18.8)	36 (62.1)	**<0.0001**
Epilepsy or seizure	16 (50.0)	9 (15.5)	**0.0005**
Facial dysmorphism	16 (50.0)	19 (32.8)	0.1083
Microcephaly	4 (12.5)	5 (8.6)	0.5571
Macrocephaly	3 (9.4)	1 (1.7)	0.0918
More than 3 dysmorphic features	9 (28.1)	3 (5.2)	**0.0022**
Other symptoms	21 (65.6)	34 (58.6)	0.5141
Family history	14 (43.8)	16 (27.6)	0.1194

SD, standard deviation; ADHD, attention deficit hyperactivity disorder; statistically significant values are highlighted in bold.

Children with positive cases were slightly older (7.54 vs 5.66 years), although the difference was not significant and only showed a trend (*p* = 0.0696). Positive cases were observed twice as often in girls (*p* = 0.0271). Among the 42 children with ASD, only 6 (14.3%) had positive or inconclusive results, which was significantly lower than in children with negative results (*p* < 0.0001). On the other hand, epilepsy or seizures were more frequently observed in children with positive cases (*p* = 0.0005). The risk of a positive genetic result in children with DD is higher in the presence of epilepsy or seizures (odds ratio – 5.4444; 95% CI 2.0176 to 14.6918; *p* = 0.0008). More than 3 dysmorphic features were also more frequently observed in positive cases (*p* = 0.0022). The risk of positive cases in children with DD is higher in the presence of more than 3 dysmorphic features (odds ratio – 7.1739; 95% CI 1.7791 to 28.9282; *p* = 0.0056). Although family history was more common in positive cases, the difference was not significant.

## Discussion

Our study demonstrates the substantial diagnostic value of WES in children with DD. The identification of pathogenic or likely pathogenic variants in nearly a third of our cohort underscores the potential of WES to uncover the genetic basis of developmental disorders that are otherwise difficult to diagnose. The correlation between genetic findings and clinical features in most cases highlights the clinical relevance of WES in guiding patient management.

Genetic testing for DD can include karyotype, microarray, Fragile X, single gene, WES, whole genome sequencing (WGS), and mitochondrial DNA testing (Bowling et al., 2017; Sun et al., 2015). WES is a method that offers broad and high-resolution identification of genetic variants and has great potential in the diagnosis of DD (Bowling et al., 2017; Stavropoulos et al., 2016). In another study, all of the aforementioned testing methods were used (Bowling et al., 2017), but WES showed the highest diagnostic value: pathogenic and likely pathogenic variants were identified in 29.8% of cases, with VUS in 11% (Bowling et al., 2017), which is consistent with our results. In our study, we performed WES on the proband only, although sequencing of proband-parent trios could have improved the diagnostic yield by identifying new mutations, as shown in other studies (Bowling et al., 2017; Brodsky et al., 2020): a pathogenic/likely pathogenic result was determined in 29.1% of trio individuals compared to 15% of singletons (Bowling et al., 2017).

WES not only ended the diagnostic odyssey for patients, allowing the avoidance of many unnecessary laboratory and instrumental tests but also influenced the treatment strategy in 34.4% of patients. In another study involving patients with epilepsy who had positive genetic results, 49.8% experienced changes in clinical management after the genetic diagnosis was established, and the majority of them (74.9%) observed improvements in outcomes (McKnight et al., 2022). For most syndromes with DD, there are no specific treatments available, although there are possibilities to address certain symptoms, such as seizures (Shelkowitz et al., 2019). Speech and language therapy, physical, occupational, behavioral therapy, and early childhood special education remain the main treatment methods for such children (Choo et al., 2019; Oberklaid and Efron, 2005). In our study, genetic testing had the greatest impact on treatment strategy, especially in patients with seizures or other conditions, including immunodeficiencies (Boyarchuk et al., 2023; Boyarchuk and Voliansla, 2023). Although early diagnosis and clinical research have enabled the development of new treatments for approximately 3% of the population with developmental disorders, including significant comorbid conditions that were previously considered incurable (Stockler-Ipsiroglu et al., 2021; Hoytema van Konijnenburg et al., 2021).

WES allowed for the identification of very rare syndromes such as *IRF2BPL*-related disease, Coffin-Siris syndrome, GLUT1 deficiency syndrome 1, and others. Notably, mitochondrial DNA abnormalities were also detected in one patient. Additionally, conditions more commonly found in populations, such as neurofibromatosis type 1 and 22q11.2 deletion syndrome, were diagnosed, where DD is not the primary symptom (Ly and Blakeley, 2019; Boyarchuk et al., 2017).

We also investigated the symptoms that had the greatest impact on positive or negative results to further enhance the diagnostic value of the method. Children with positive WES results were more likely to have epilepsy or seizures and more than three dysmorphic features. A comparison of baseline characteristics between children with positive and negative findings revealed a trend towards an older age at the time of a positive result (*p* = 0.0271). This is likely because, in older children, more clinical manifestations of the disease are present, making it easier to correlate the identified variants with clinical signs.

In children with ASD, a significantly lower rate of positive or inconclusive results was observed. Pathogenic and likely pathogenic variants (*PTEN* and *NF1*) were identified in two children, who also presented with other dysmorphias. According to another study, variants in the *PTEN* gene were detected in 17% of children with ASD and macrocephaly (Butler et al., 2005). Therefore, *PTEN* gene sequencing should be considered in all patients with autism and macrocephaly (head circumference >2.5 SD) (Schaefer and Mendelsohn, 2008). Neurofibromatosis (*NF1* and *NF2* genes) is reported as one of the main causes of single-gene disorders associated with ASD (Genovese and Butler, 2023), along with tuberous sclerosis, X-linked Rett syndrome (*MECP2* gene), and fragile X syndrome (*FMR1* gene).

In two children with ASD from our cohort, VUS (in *DPF2* and *DEAF* genes) were detected, and in one child suspected of having Rett syndrome, a 15q11.2q12q13.1 duplication syndrome was identified, which correlated with the clinical data. One child had positive secondary findings (*PALB2*), which is associated with an increased susceptibility to breast cancer. Overall, ASD is considered a multifactorial disorder, with genetics playing a significant role, especially considering the presence of similar conditions in the family, which, according to various authors, account for 60%–90% (Genovese and Butler, 2023). The relevance of genetic influences is also indicated by the significant increase in the number of scientific publications on this topic in PubMed, although most of the studies are of a review nature. To date, more than 800 genes have been identified that may be associated with ASD. The most common chromosomal abnormalities in children with ASD are microdeletion or duplication syndromes of 2q37, 7q35, 15q11-13, 22q11, 22q13, and 18q (Butler et al., 2005; Schaefer and Mendelsohn, 2008). Overall, up to 50% of genetic causes of ASD are due to cytogenetic defects (Genovese and Butler, 2023).

It is believed that the effectiveness of genetic tests in ASD ranges from 6% to 15% (Schaefer and Mendelsohn, 2008), while it is reported that the effectiveness of WES ranges from 9% to 30% (Boyarchuk et al., 2017), which aligns with the results of our study, where variants compatible with the clinical manifestations of ASD were identified in 5 out of 42 (11.9%) children. Researchers also note that the presence of additional signs such as psychiatric conditions, ataxia, or paraplegia increases the chances of a positive result (Rossi et al., 2017). Another study showed that using chromosomal microarray analysis (CMA) and WES improved the diagnosis of ASD (Tammimies et al., 2015), although in three of our patients, microdeletion and duplication syndromes were identified using WES.

Other factors also play a role in the development of ASD, particularly epigenetic ones (Masini et al., 2020). Therefore, in children with ASD, it is always necessary to assess environmental factors, such as the course of pregnancy, including maternal nutrition, infectious diseases during pregnancy, the impact of medications, toxins, smoking, and other harmful factors on the fetus, maternal age, *etc.*

Raising awareness among physicians, medical staff, and medical students about rare genetic diseases, along with creating opportunities for their diagnosis, will enable timely diagnosis and, in many cases, improve the quality and length of patients' lives (Boyarchuk et al., 2019; Boyarchuk et al., 2018).

### Strengths and limitations

Thus, the comprehensive use of WES for diagnosing DD allowed us to identify a range of genetic variants, including very rare syndromes. Additionally, we identified deletion and duplication syndromes, as well as mitochondrial DNA disorder. The study also provided valuable insights into the clinical utility of WES in improving diagnostic yield, particularly in cases with epilepsy or multiple dysmorphic features. Furthermore, the findings highlighted the importance of early genetic testing in guiding clinical management and treatment decisions, which could lead to improved patient outcomes.

The study has some limitations. Firstly, regarding the interpretation of VUS, we conducted only careful clinical correlation without performing additional tests due to limited resources. While WES provided a definitive diagnosis in many cases, the interpretation of some findings remained challenging due to the lack of clear phenotypic-genotypic correlations at the time of testing. The use of WES trio could have improved the detection of new variants or other genes that play a role in the development of DD.

## Conclusion

WES is a powerful diagnostic tool for uncovering the genetic basis of DD in children. Variants consistent with clinical manifestations were detected in 30% of patients with DD, including 11.9% of children with ASD. The method also allowed the identification of microdeletion and duplication syndromes, as well as mitochondrial DNA disorders. The presence of epilepsy and more than three dysmorphic features enhances the diagnostic value of WES in diagnosing DD. Genetic diagnosis led to a revision of the diagnosis or management plan in 34.4% of positive cases. Our findings suggest that WES should be considered in the diagnostic workup of pediatric patients with unexplained developmental delays, as it has the potential to significantly impact patient care.

## Data Availability

The datasets presented in this study can be found in online repositories. The names of the repository/repositories and accession number(s) can be found in the article/supplementary material.
