# Supplementary material for: Exome sequencing in 90 children with developmental delay: a single-center experience
Source: Front Genet. 2024 Nov 29;15:1505254. doi: 10.3389/fgene.2024.1505254 (PMC11638168; doi:10.3389/fgene.2024.1505254)
Supplement: Supplementary file 1 [file Table1.docx]

**Table S1**. Distribution of identified variants and their clinical relevance

| Patient/  gender | Age | Genes | Variant | Classification | Zygosity | Inheritance | Variant associated diagnosis | Impact on treatment |
| --- | --- | --- | --- | --- | --- | --- | --- | --- |
|  | **Positive results** (pathogenic or likely pathogenic) | | | | | | | |
| 1 / M | 2 | *ZMYND11* | c744_745del, p.Cys249SerfsTer2 | P | Het | AD | Intellectual disability | No |
| 2 / F | 4 | *ZMYND11* | c.6972T>A Protein: NP_001357029.1:p.? | LP | Het | AD | Intellectual developmental disorder, autosomal dominant 30, OMIM: 616083 | No |
| 3 / M | 2 | *AIRE* | c769C>T | P | Hom | AR | APECED (APS1) | Yes |
| 4 / M | 2 | *KCNQ2* | c593G>A, p.Arg198Gln | P | Het | AD | Epileptic encephalopacy, early infantile, 7 | Yes |
| 5 / M | 2 | *NF1* | c.3916C>T, p.Arg1306Ter | P | Het | AD | Neurofibromatosis - Noonan syndrome | Yes |
| 6 / F | 4 | *NF1* | c.3436G>T, p.Val1146Phe | LP | Het | AD | Neurofibromatosis, type 1 | Partly |
| 7 / M | 4 | *GLB2* | c.35del, p.Gly12ValfsTer2 | P | Hom | AR | Deafness, autosomal recessive 1A | Yes |
| 8 / F | 0.2 | 15q25.1q26.3 duplication |  | P | Het |  | 15q25.1q26.3 duplication | No |
| 9 / M | 2 | *CHD2* | c.245C>T, p.Arg1152Trp | LP | Het | AD | Developmental and epileptic encephalopathy 94 | Yes |
| 10 / M | 9 | *IRF2BPL* | c.1489C>T, p.Gln497Ter | LP | Het | AD | Neurodevelopmental disorder with regression, abnormal movements, loss of speech, and seizures | Partly |
| 11 / F (sybling) | 12 | *IRF2BPL* | c.1489C>T, p.Gln497Ter | LP | Het |  |  | Partly |
| 12 / M | 17 | *KCNJ11* | c.405 dup, p.Arg136AlafsTer5 | LP | Het | AD | Diabetes permanent neonatal 2, with or without neurologic features | Partly |
| 13 / F | 5 | *PTEN* | c493-1G>T, p.? | LP | Het | AD | Macrocephaly/autism syndrome, IEI | Partly |
| 14 / F | 5 | *EIF3F* | c.694T>G, p.Phe232Val | P | Hom | AR | Intellectual disability, AR67 | No |
| 15 / F | 13 | *SATB1* | c1588G>A, p.Glu530Lys | P | Het | AD | Kohlscutter-Tonz syndrome-like | Partly |
| 16 / F | 9 | *ATP1A3* | c.2415C>A, p.Asp805Glu | LP | Het | AD | Developmental and epileptic encephalopathy | Yes |
| 17 / F | 2 | *APOE* | c.538C>T, p.Arg180Cys | LP | Het | AD | Hyperlipoproteinemia, type III | Yes |
| 18 / M | 16 | *SMC3 ;*  *MT-TL1* | c.707G>A,  p.Arg236His  m.3243A>G | LP | Het  Heteroplasmic | AD | Cornelia de Lange syndrome 3;  MELAS | Partly |
| 19 / M | 13 | *DGCR2, ESS2* + 42 genes |  | P |  |  | 22q11.2 Deletion Syndrome | Yes |
| 20 / F | 5 | *SLC2A1* | c781G>T, p.Glu261Ter | LP | Het | AD | GLUT1 deficiency syndrome 1, infantile onset, severe | Partly |
| 21 / F | 13 | *BBS7* | c.712_715del,  p.Arg238GlufsTer59 | P | Hom | AR | Bardet-Biedl syndrome 7 | Partly |
|  | Positive results (secondary findings) | | | | | | | |
| 22 / M | 16 | *SCN5A* | с.2707А>G, p.Met903Val | LP | Het | AD | Long QT syndrome 3, Brugada syndrom1 | Partly |
| 23 / F | 12 | *BRCA2* | pSer3366AsnfsTer5 | LP | Het | AD | Breast-ovarian cancer, familial, 2 | Partly |
| 24 / M | 4 | *KCNQ1* | с.590C>T, p.Pro197Leu | LP | Het | AD | Long QT syndrome 1 | Partly |
| 25 / M | 5 | *PALB2* | C,1655del,  p.Gln552ArgfsTer9 | LP | Het | AD | Breast cancer, susceptibility to (OMIM: 114480) | Partly |
|  | **Inconclusive** | | | | | | | |
| 26 / F | 16 | *CHD2* | c.5053C>T, p.Arg1685Cys | VUS | Het | AD | Epileptic encephalopacy, childhood onset | Yes |
| 27 / F | 4 | *KDM5C* | c.1762C>T, p.Gln588Ter | LP | Het | AD | Intellectual disability, X-linked, syndromic, Claes-Jensen type | Partly |
| 28 / M | 16 | *SMARCA4* | c.265T>C, p.Met886Thr | VUS | Het | AD | Coffin-Siris syndrome 4 | Yes |
| 29 / M | 6 | *DPF2* | c.976G>A, p.Glu326Lys | VUS | Het | AD | Coffin-Siris syndrome 7 (OMIM: 618027) | Partly |
| 30 / F | 13 | 15q11.2q12q13.1 duplication |  |  | Het |  | 15q11.2q12q13.1 duplication | Partly |
| 31 / M | 4 | *DEAF1* | c.644A>G,  p.Tyr215Cys | VUS | Het | AD | Vulto-van Silfout-de Vries syndrome, OMIM: 615828 | No |
| 32 / M | 4 | *TNFRSF13B* | c.542C>A,  p.Ala181Glu | P | Het | AR, AD | Common variable immunodeficiency, 2 (OMIM: 240500) | Yes |

VUS- Variant of uncertain significance, LP *-* Likely pathogenic, P- Pathogenic
